# Supplementary material for: Inflammation of Dry Eye Syndrome: A Cellular Study of the Epithelial and Macrophagic Involvement of NFAT5 and RAGE
Source: Int J Mol Sci. 2023 Jul 4;24(13):11052. doi: 10.3390/ijms241311052 (PMC10341472; doi:10.3390/ijms241311052)
Supplement: Supplementary file 1 [file ijms-24-11052-s001.zip › ijms-2429930-supplementary.pdf]

Supplementary data

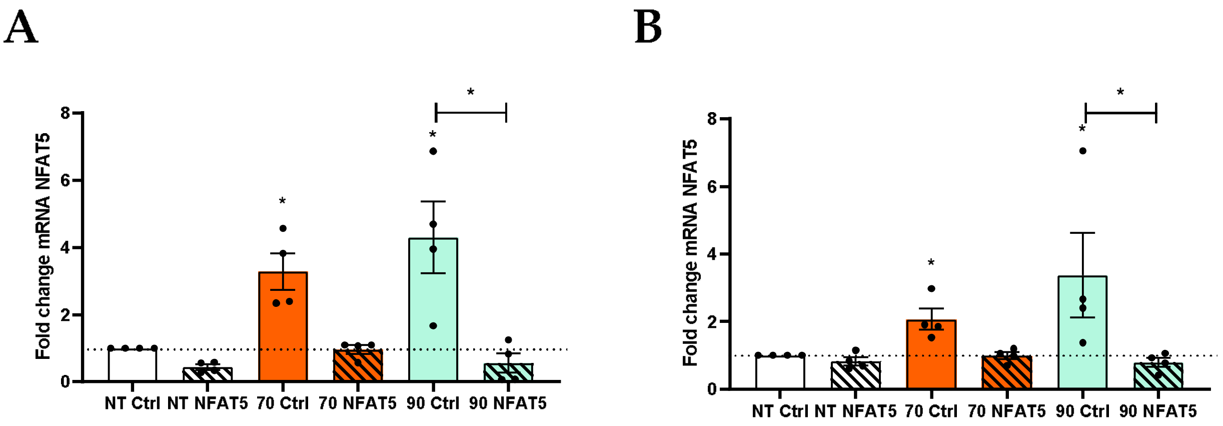

Figure S1. Validation of siRNA NFAT5. After transfection with siRNA against NFAT5 or siRNA control (Ctrl), cells were exposed to a hyperosmolar medium (70 mM NaCl [orange] or 90 mM NaCl [green]) for 24h and analyzed for mRNA expression of NFAT5 by RT-qPCR in **(A)** a Wong-Kilbourne derivative of Chang conjunctival (WKD) cell line and **(B)** a human corneal epithelial (HCE) cell line. The experiment was repeated four times (n=4). Results are expressed as a fold change between treated conditions and non-treated (NT Ctrl) and were analyzed by ANOVA statistical test (Kruskal-Wallis) followed by Dunn's post hoc test: * $p = 0.05$. The horizontal line corresponds to the value of the non-treated cells reported as 1.

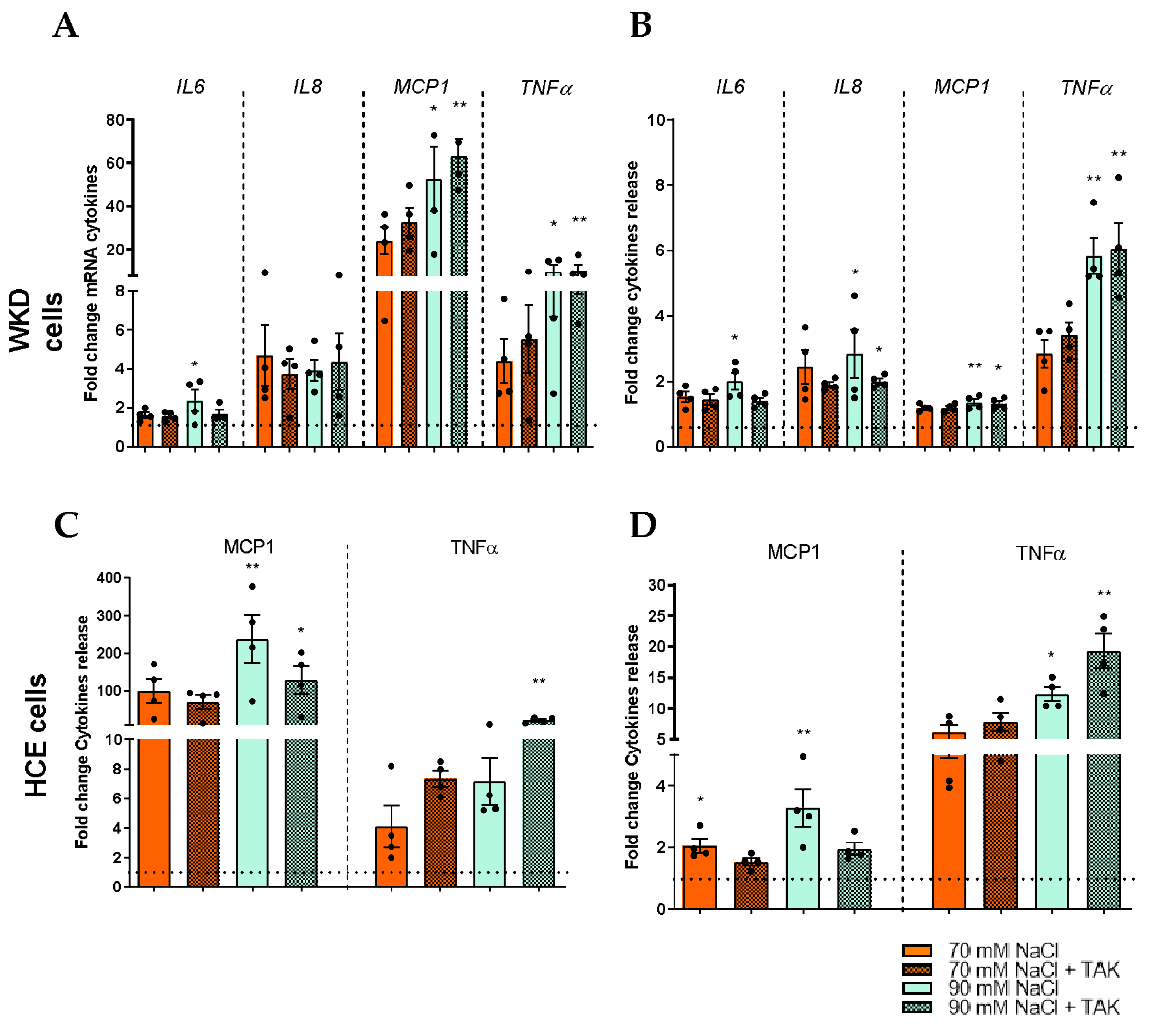

Figure S2 : Implication of TLR4 on the cytokines release of epithelial cells. Cells were exposed to a hyperosmolar medium (70 mM NaCl [orange] or 90 mM NaCl [green]), with or without TAK (TLR4 inhibitor) for 24h and **(A)** then analyzed for mRNA expression of cytokines (IL6, IL8, TNF*α* and MCP1) by RT-qPCR and **(B)** exposed 48 h with hyperosmolarity for the cytokines protein release analyzes in a Wong-Kilbourne derivative of the Chang conjunctival (WKD) cell line. **(C)** The mRNA expression of cytokines and **(D)** protein release were analyzed in a human corneal epithelial (HCE) cell line.The experiment was repeated four times (n=4). Results are expressed as a fold change between treated conditions and non-treated (NT) and were analyzed by ANOVA statistical test (Kruskal-Wallis) followed by Dunn's post hoc test: * $p = 0.05$ and ** $p = 0.01$. The horizontal line corresponds to the value of the non-treated cells reported as 1.

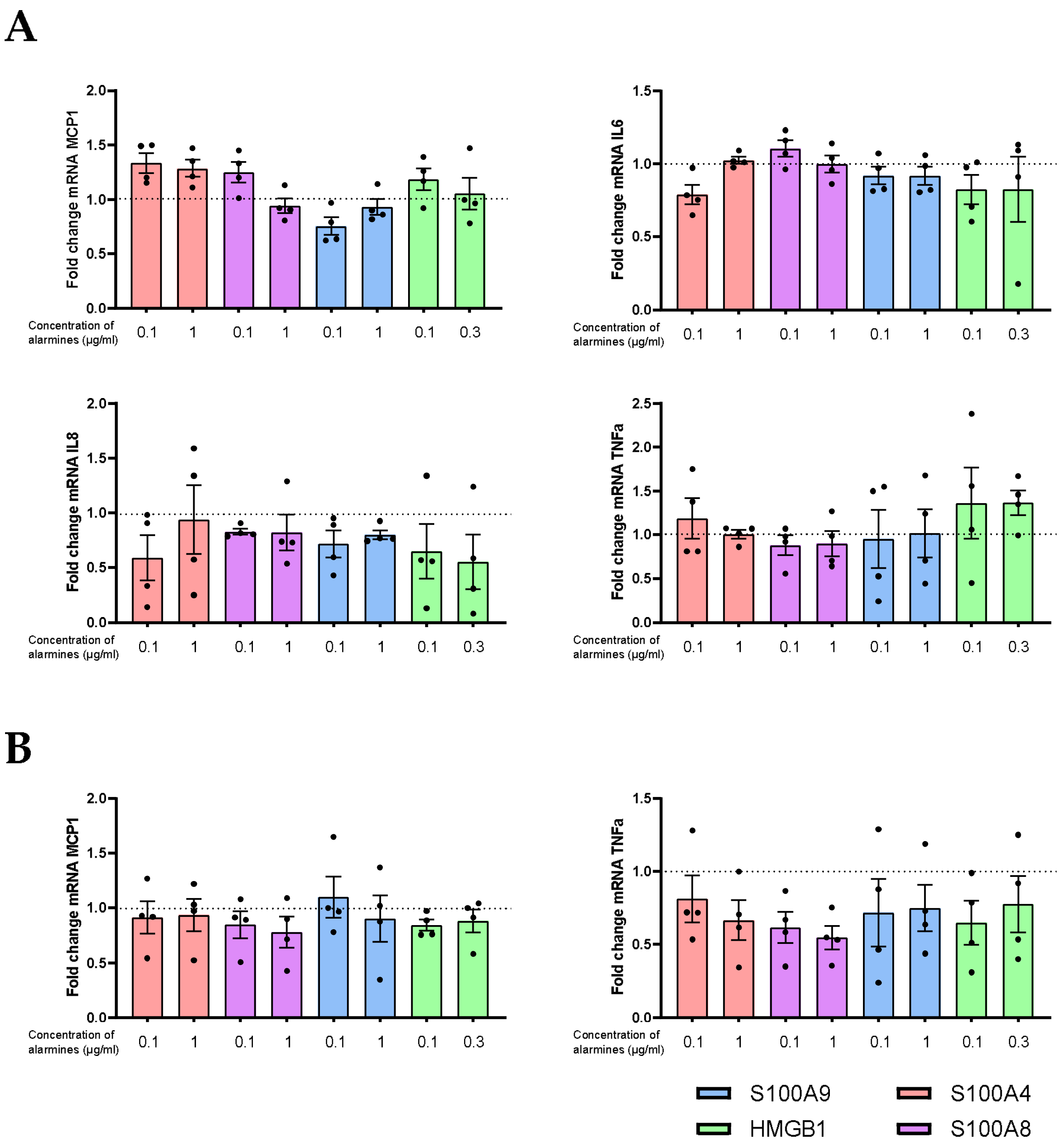

Figure S3 : Action of DAMPs on epithelial cells expression of cytokines mRNA. Epithelial cells were exposed to normal medium with recombinant proteins (S100A4, S100A8 or S1009 at 0.1 and 1 µg/ml, or HMGB1 at 100 and 300 ng/ml) for 24h and then analyzed for mRNA expression of IL6, IL8, TNFα and MCP1 by RT-qPCR in **(A)** a Wong-Kilbourne derivative of Chang conjunctival (WKD) cell line and (B) a human corneal epithelial (HCE) cell line. The experiment was repeated four times (n=4). Results were expressed as a fold change between treated conditions and non-treated (NT) and were analyzed by ANOVA statistical test (Kruskal-Wallis) followed by Dunn's post hoc test. The horizontal line corresponds to the value of the non-treated cells reported as 1.
